# Quantification of indocyanine green fluorescence angiography in colorectal surgery: a systematic review of the literature

**DOI:** 10.1007/s00464-025-11621-8

**Published:** 2025-02-28

**Authors:** Philip D. McEntee, Ashokkumar Singaravelu, Cathleen A. McCarrick, Edward Murphy, Patrick A. Boland, Ronan A. Cahill

**Affiliations:** 1 UCD Centre for Precision Surgery, UCD, 47 Eccles Street, Dublin 7, Dublin, Ireland; 2 Department of Surgery, Mater Misericordiae University Hospital, Dublin, Ireland

**Keywords:** Colorectal surgery, Near infrared laparoscopy, Indocyanine green, Fluorescence angiography, Quantification

## Abstract

**Background:**

Indocyanine green fluorescence angiography (ICGFA) during colorectal surgery associates with reduced post-operative anastomotic complication rates. Because its interpretation is subjective, quantification has been proposed to address inter-user variability. This study reviews the published literature regarding ICGFA quantification during colorectal surgery with a focus on impactful clinical deployment.

**Methods:**

A systematic review was performed of English language publications regarding clinical studies of ICGFA quantification in colorectal surgery in PubMed, Scopus, Web of Science and Cochrane Library on 29th August 2024, updated to 18th November 2024, following PRISMA guidelines. Newcastle Ottawa scale (NOS) was used to assess quality.

**Results:**

A total of 1428 studies were screened with 22 studies (1469 patients) selected. There was significant heterogeneity of ICGFA methodology, quantification methods and parameter selection and only three studies were NOS “high” quality. Extracorporeal application was most common. Four studies (154 patients) conducted real-time ICGFA analyses (others were post hoc) and four utilised artificial intelligence methods. Eleven studies only included patients undergoing left-sided resection (six focusing specifically on rectal resections). Only one study employed the quantification method to guide intra-operative decision-making regarding colonic transection. Twenty-six different perfusion parameters were assessed, with time from injection to visible fluorescence and maximum intensity the most commonly (but not only) correlated parameters regarding anastomotic complication (*n* = 18). Other grounding correlates were tissue oxygenation (*n* = 3, two with hyperspectral imagery), metabolites (*n* = 2) and surgeon interpretation (*n* = 5).

**Conclusion:**

Quantification of the ICGFA signal for colorectal surgery is feasible but has so far seen limited academic advancement beyond feasibility.

Colorectal surgery remains the mainstay of treatment for both benign and malignant colorectal disease. One of its most critical complications is anastomotic leakage (AL), with recent randomised controlled trials (RCTs) reporting an incidence of up to 23% following anterior resection of the rectum [1–4]. AL is associated with increased secondary complications, mortality, hospital length of stay and costs and poorer oncological outcomes [5–11].

Alongside many risk factors for AL following colorectal resectional surgery such as smoking, male gender, obesity, age, diabetes, neoadjuvant therapy, tumour location [12–15], ensuring appropriate perfusion of the anastomosis is a key step in mitigating AL [16]. Traditionally, surgeons have assessed bowel perfusion using various parameters such as palpable pulsation of the mesentery, colonic discolouration and active bleeding at the resection margin [12], however, these factors have been found to be subjective and unreliable [17].

Recent large scale RCTs are now concluding that indocyanine green fluorescence angiography (ICGFA) provides a rapid and reliable intra-operatively bowel perfusion assessment [3, 18–23] by presenting near-infrared (NIR) imagery for the surgeon to interpret, that may become a new standard of care for restorative colorectal surgery. However potential barriers to its widespread adoption remain including its subjective nature, with significant inter-user variability [24–26] evident, and associated learning curve. Computer vision methods of ICGFA quantification have been proposed as a means of adding robustness to the technique and so it is now timely to consider their merit in overcoming interpretation issues in the near-term and so be part of any technology roll-out. Here we review the state of the art via published literature regarding ICGFA signal quantification in colorectal surgery with a focus on impactful clinical deployment.

## Methods

### Search strategy

This review did not meet the criteria for a systematic review as per Prospero criteria as there was no unifying, single, primary outcome [27] and so a systematic literature search was carried out according to PRISMA-Search checklist guidelines and recommendations [28]. PubMed, Scopus, Web of Science and Cochrane Collaboration database were searched for relevant publications initially from inception to 29th August 2024 and then updated to 18th November 2024 using the following Medical Subject Heading (MESH) search terms: “colorectal” (includes “colon” and “rectum”), “bowel”, “anastomosis”, “anastomotic leak”, “outcome”, “perfusion”, “assessment”, “evaluation”, “fluorescence”, “angiography”, “indocyanine green”, “quantification”, “computation”. All terms were “exploded” to include subheadings and the truncation symbol (*) used as appropriate. The Boolean operator “OR” was used within concepts, whilst “AND” was used to link concepts. Titles and abstracts were then screened by two reviewers (PMcE and AS). Additionally, reference lists in relevant publications and Google Scholar were screened for other relevant publications.

### Study selection

For inclusion in analysis, studies had to meet the following criteria:

- Publication of a clinical study in patients undergoing colorectal surgery for benign or malignant disease
- ICGFA use for assessment of bowel perfusion
- A method to quantify the fluorescence imagery
- Full text available in English

Studies were excluded if any of the following criteria were met:

- No human patients (e.g. animal studies)
- Publication types such as systematic reviews, meta-analyses, guidelines, conference abstracts.

### Outcome of interest

The studies included were compared based on the methodology used, the ICG dosage, the NIR camera system and the quantification software. Types of operations performed and timing of ICG administration were recorded. Measured parameters, endpoints of the studies, the rate of change of mind based on fluorescence imaging and the anastomotic complication rates were summarised.

### Data extraction

The selection process was assisted by an online tool (Rayyan®) [29].Two reviewers (PMcE and AS) independently examined studies according to the predefined strategy and criteria. Each extracted and recorded separately the title and publication details in addition to population characteristics. Databases were reviewed and compared at the end of the reviewing process to limit selection bias, remove duplicates and a third reviewer (PB) was used to clarify any disputes.

### Quality assessment

The quality assessment was performed by two authors (PMcE and AS) independently. Studies were assessed using the Newcastle Ottawa Scale (NOS) [30]. In the case of any disputes, a third author (PB) was consulted to clarify. Traffic light plots to present NOS assessments were created using the “robvis” package in RStudio 2023.12.0 [31].

## Results

### Study characteristics

**Fig. 1:**
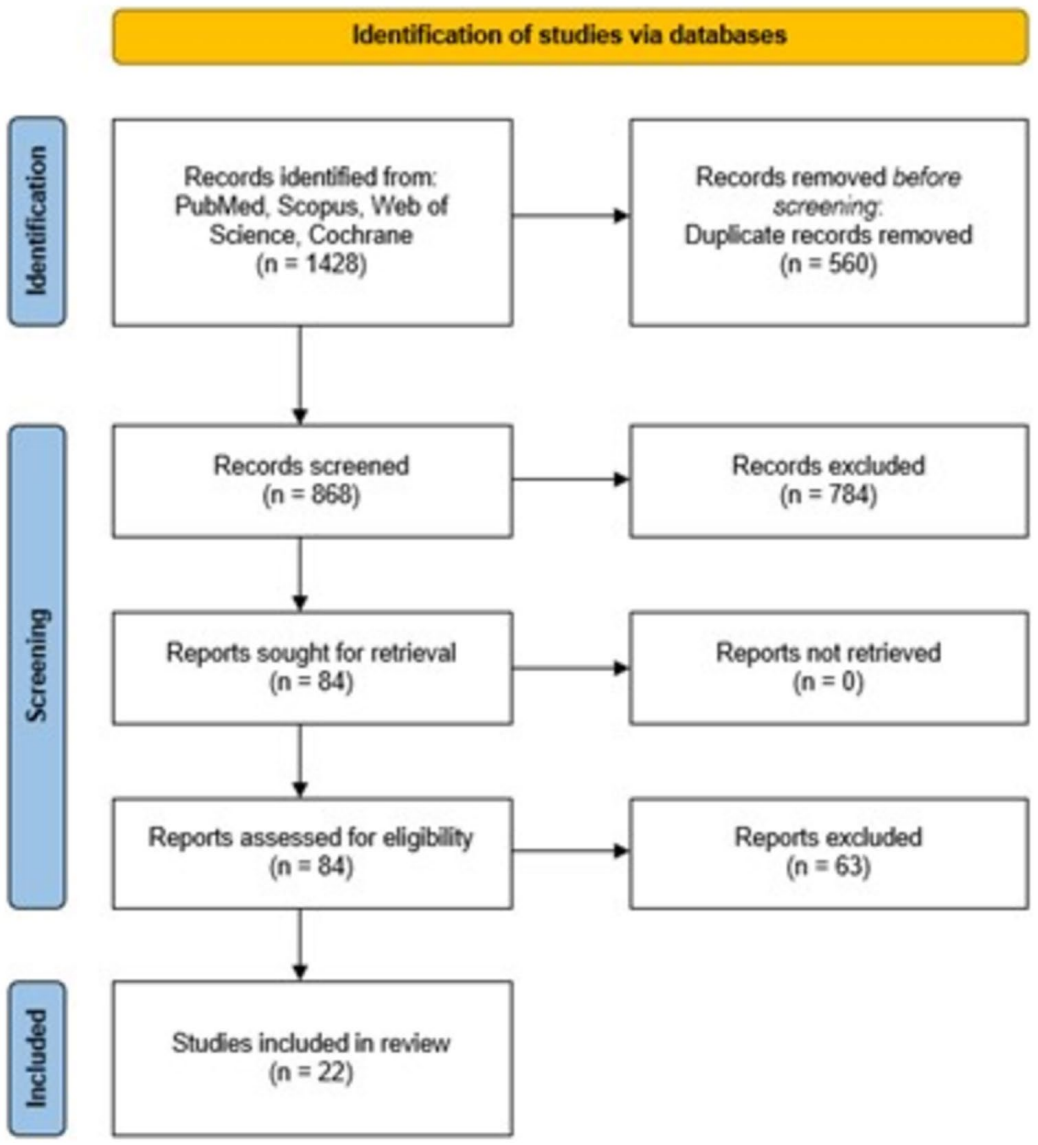
PRISMA flowchart of study selection

**Fig. 2:**
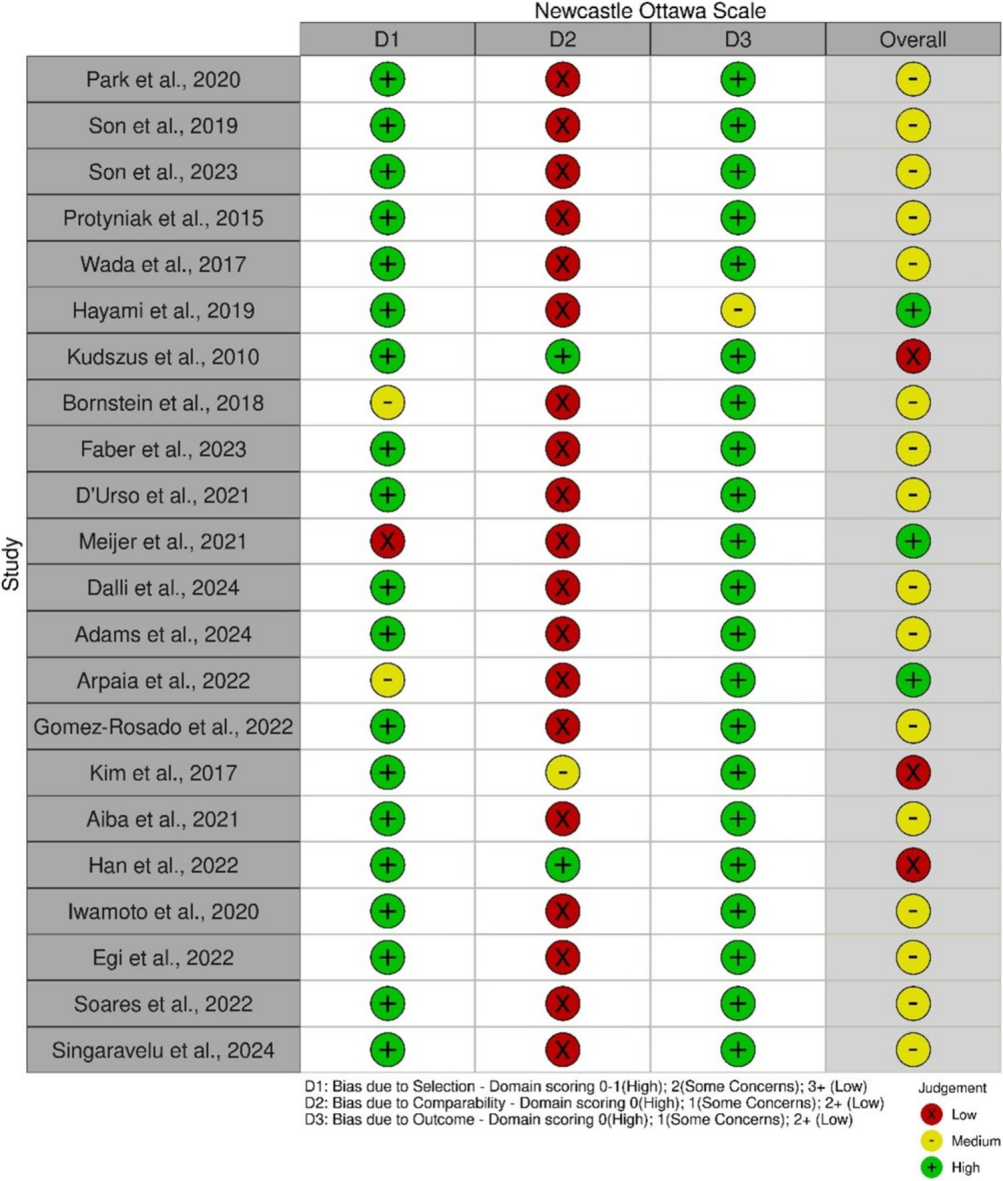
Traffic light plot for Newcastle Ottawa Score results

The initial search yielded a total of 1428 studies, of which 84 met the criteria for full review, with 22 being finally included in this study as depicted in the PRISMA flow diagram (Fig. 1). All these studies were published after 2010, with fifteen (68.1%) being published from 2020 onwards. Together, the 22 studies included quantitative ICGFA analysis data from a total of 1469 patients, with the number of patients in each study ranging from 4 to 301. In four studies (154 patients) real-time analysis of the fluorescence signal was performed with the remainder (1315 patients) utilising post hoc video recording analysis (Table 1) [12, 32–52]. The senior corresponding author was a surgeon in twenty-one studies with the other one being coordinated by a scientist [44]. NOS evaluated 16 studies as moderate quality with three each being high and low quality. (Fig. 2).

**Table 1:** Study and patient characteristics. NR not recorded

Publication	Patient number	Study period	Operations	Analysis	Senior author
Park et al. (2020)	65	August 2018–May 2019	Anterior resection/low anterior resection	Post hoc	Surgeon
Son et al. (2019)	86	July 2015–December 2017	Anterior resection/low anterior resection	Post hoc	Surgeon
Son et al. (2023) + Hyperspectral Imaging	68	January 2021–December 2022	Sigmoid/rectal resection	Real-time	Surgeon
Protyniak et al. (2015)	77	June 2013–June 2014	Multiple sites within colon and rectum	Post hoc	Surgeon
Wada et al. (2017)	112	August 2013–April 2016	Left-sided colorectal resections	Post hoc	Surgeon
Hayami et al. (2019)	22	December 2014–September 2015	Multiple sites within colon and rectum	Post hoc	Surgeon
Kudszus et al. (2010)	201	2003–2008	Multiple sites within colon and rectum	Post hoc	Surgeon
Bornstein et al. (2018)	49	NR	Multiple sites within small bowel, colon and rectum	Post hoc	Surgeon
Faber et al. (2023)	20	NR	Multiple sites within colon and rectum	Post hoc	Surgeon
D'Urso et al. (2021)	22	January 2017–November 2018	Left-sided colorectal resections	Real-time	Surgeon
Meijer et al. (2021)	4	NR	Varied operations with mesenteric resection	Post hoc	Surgeon
Dalli et al. (2024)	23	NR	Multiple sites within colon and rectum	Post hoc	Surgeon
Adams et al. (2024)	89	August 2021–June 2022	Multiple sites within colon and rectum	Post hoc	Surgeon
Arpaia et al. (2022)	11	NR	Multiple sites within colon and rectum	Real-time	Scientist
Gomez-Rosado et al. (2022)	70	July 2020–February 2021	Multiple sites within colon and rectum	Post hoc	Surgeon
Kim et al. (2017)	310	2013–2016	Low and ultra-low anterior resections	Post hoc	Surgeon
Aiba et al. (2021)	110	September 2017–January 2020	Multiple sites within colon and rectum	Post hoc	Surgeon
Han et al. (2022)	22	February 2020–July 2020	Left-sided colorectal resections	Post hoc	Surgeon
Iwamoto et al. (2020)	25	July 2016–June 2017	Anterior resection	Post hoc	Surgeon
Egi et al. (2022)	12	January 2021–March 2021	Left-sided colorectal resections	Post hoc	Surgeon
Soares et al. (2022)	18	May 2019–November 2019	Multiple sites within colon and rectum	Post hoc	Surgeon
Singaravelu et al. (2024)	53	NR	Multiple sites within colon and rectum	Real-time	Surgeon

### ICG protocol, visualisation and software

Most (*n* = 20) studies assessed ICGFA of the colonic segment intended for anastomosis prior to anastomotic formation, with one other study assessing ICGFA both before and after [12], and one after only anastomosis [37]. Eleven studies included only patients undergoing left-sided resection, with six of these focusing specifically on rectal resections (576 patients). One of the included rectal-only studies assessed only the distal rectal stump after resection of the specimen [49]. The remaining eleven studies included patients undergoing resectional surgery for a variety of benign and malignant pathologies throughout the colon, rectum and small bowel. The dose of ICG given varied significantly also. Eight of the studies administered a weight-dependent dose of ICG, with ten studies using a fixed dose regimen (four studies did not report dosing). Various camera systems were used with manufacturers including Stryker [32, 34, 42, 43, 49–52], Karl Storz [33, 37, 40, 47], Novadaq [35, 38], Hamamatsu Photonics [36], Pulsion Medical System [12], Quest Medical Imaging [39, 41], Olympus [44, 48], Medtronic [45], Intuitive [46, 48] and Arthrex [52]. Thirteen studies performed extra-corporeal ICG assessment of the region of interest, whilst two studies performed intra-corporeal assessment, with six studies using a mixture (one study did not report this). Of the six studies examining rectal resections only, two each performed ICGFA assessment extra-corporeally, intra-corporeally or as a mixture. Eighteen of the studies reported an image capture protocol, with twelve reporting a specified length of time, ten reporting a fixed distance between the camera and the tissue of interest and five reporting turning off the background lights for extracorporeal assessment. ICGFA signal quantification was carried out using a variety of commercially available [36, 37, 49] or bespoke [32, 38, 42, 52] software, however none of the latter seem to have been made available yet online for use by others (Table 2).

**Table 2:** ICG protocol, visualisation, software and perfusion parameters

Paper	Timing (relative to anastomosis)	Dosage	Camera system	Intra- or extra-corporeal ICG assessment	Image capture Protocol	Quantification system	Parameters used
Park et al. (2020)	Prior	0.2 mg/kg	1588 AIM, Stryker	Mixed	Camera 6–8 cm from tissue, for 2 min after injection	MATLAB 2019, MathWorks	T_max_, T_1/2max_, TR, F_max_, RS
Son et al. (2019)	Prior	0.25 mg/kg	IMAGE1 S, Karl Storz	Mixed	For 2 min after injection, up to 5 min if poor perfusion	Tracker 4.97, Douglas Brown, Open Source	T_max_, T_1/2max_, TR, F_max_, RS
Son et al. (2023)	Prior	0.2 mg/kg	1588 AIM, Stryker	All extra-corporeal	Camera 4–5 cm from tissue, lights off, for 2 min after injection	ICG Analyzer Program 8.0, Pusan National University	T_max_, T_1/2max_, TR, F_max_, RS
Protyniak et al. (2015)	Prior	NR	SPY Elite Imaging System, Novadaq	Mixed	For 60 s after visible fluorescence	SPY-Q, Stryker	T_max_
Wada et al. (2017)	Prior	5 mg	PDE-neo System, Hamamatsu Photonics	All extra-corporeal	Camera 15 cm from tissue, lights off	ROIs, Hamamatsu Photonics	T_max_, T_1/2max_, F_max_, RS
Hayami et al. (2019)	Post	5 mg	D-light P system, Karl Storz	All extra-corporeal	Camera 20 cm from tissue, for 5 min after injection	ROIs, Hamamatsu Photonics	T_0_, T_max_, T_1/2max_, F_max_, RS
Kudszus et al. (2010)	Pre and post	0.2–0.5 mg/kg	IC-View, Pulsion Medical System	All extra-corporeal	Tripod-mounted camera, lights off, loop of small intestine as reference	IC-Calc®	NR
Bornstein et al. (2018)	Prior	NR	SPY Elite Imaging System, Novadaq	All extra-corporeal	For 113–128 s after injection	Modified Comprehensive Angiographic and Perfusion Analysis platform	F_max_ and ‘Timing’
Faber et al. (2023)	Prior	5 mg	Quest Spectrum 2.0, Quest Medical Imaging	All extra-corporeal	Camera 30 cm above tissue, for 5 min after injection, 90 degrees to tissue, standardised camera settings	Quest Research Framework, Quest Medical Imaging	T_max_, F_max_, Ingress Slope, RS, Normalised Slope, Egress Slope, AUC_30/60/120/180s_
D'Urso et al. (2021)	Prior	0.2 mg/kg	D-light P system, Karl Storz	All extra-corporeal	NR	Fluorescence-based enhanced reality (FLER)	T_max_
Meijer et al. (2021)	N/A	5 mg	Quest Spectrum, Quest Medical Imaging	All extra-corporeal	NR	Mevislab, MeVis Medical Solutions AG	T_max_, T_1/2max_, TR, F_max_, F_1/2max_ RS, RS10s
Dalli et al. (2024)	Prior	0.1 mg/kg	Pinpoint, Stryker	Mixed	4 min from visible fluorescence	IBM Tracker + MATLAB	T_0_, T_max_, T_1/2max_, TR, F_max_, F_50s_, F_100s_, Downslope, Downslope_50s_, Downslope_100s_, COM
Adams et al. (2024)	Prior	NR	SPY-PHI QP, Stryker	All extra-corporeal	2 min from visible fluorescence, bowel 10 cm proximal to resection margin as reference	SPY-PHI QP, Stryker	T_max_, F_max_
Arpaia et al. (2022)	Prior	NR	Visera Elite, Olympus	NR	NR	Minimum Output Sum of Squared Error (MOSSE) tracker. Python 2.7—TensorFlow, Keras, OpenCV	NR
Gomez-Rosado et al. (2022)	Prior	7.5 mg	VisionSense VS Iridium, Medtronic	Mixed	Camera 15 cm from tissue, standardised camera lens, lights off	Elevision IR, Medtronic	T0, T_max_, T_max_ + T0, F_max_, F_maxroi_, RS
Kim et al. (2017)	Prior	10 mg	Da Vinci Firefly, Intuitive	All extra-corporeal	According to manufacturer’s instructions	Color Standards, Microsoft PowerPoint	T_0mesocolon_, T_0colon_, F_max_ (grades 1–5)
Aiba et al. (2021)	Prior	0.1 mg/kg	OPAL1, Karl Storz	All extra-corporeal	Camera 5 cm above tissue, lights off	NA	T_0_
Han et al. (2022)	Prior	7.5 mg	Visera Elite, Olympus or Da Vinci Firefly, Intuitive	All intra-corporeal	2 min after visible fluorescence, injection at start of case for baseline	HSL Video Analyzer, Dr. Park's Software Lab	T_max_, T_1/2max_, TR, F_min_, F_max_, RS, T_plateau_ F_plateau_, Slope_plateau_,
Iwamoto et al. (2020)	Prior	7.5 mg	Pinpoint, Stryker	All intra-corporeal	Camera 5 cm from tissue, for 5 min after injection	ROIs, Hamamatsu Photonics	T_0_, T_max_
Egi et al. (2022)	Prior	5 mg	Pinpoint, Stryker	All extra-corporeal	NR	NA	T_0_
Soares et al. (2022)	Prior	10 mg	Pinpoint, Stryker	All extra-corporeal	Tripod-mounted camera	NR	F_max_
Singaravelu et al. (2024)	Prior	01.mg/kg	Pinpoint/Stryker 1688, Styker and Synergy Vision, Arthrex	Mixed	90 s after ICG injection with indication of actual transection site	MATLAB R2024a, MathWorks	T_max_, T_1/2max_, F_max_, RS

*NR* not recorded, *TR* time ratio, *RS* relative slope, *AI* artificial intelligence, *T0* time to visible fluorescence, *AUC* area under curve, *COM* centre of mass

### Perfusion parameters

**Fig. 3:**
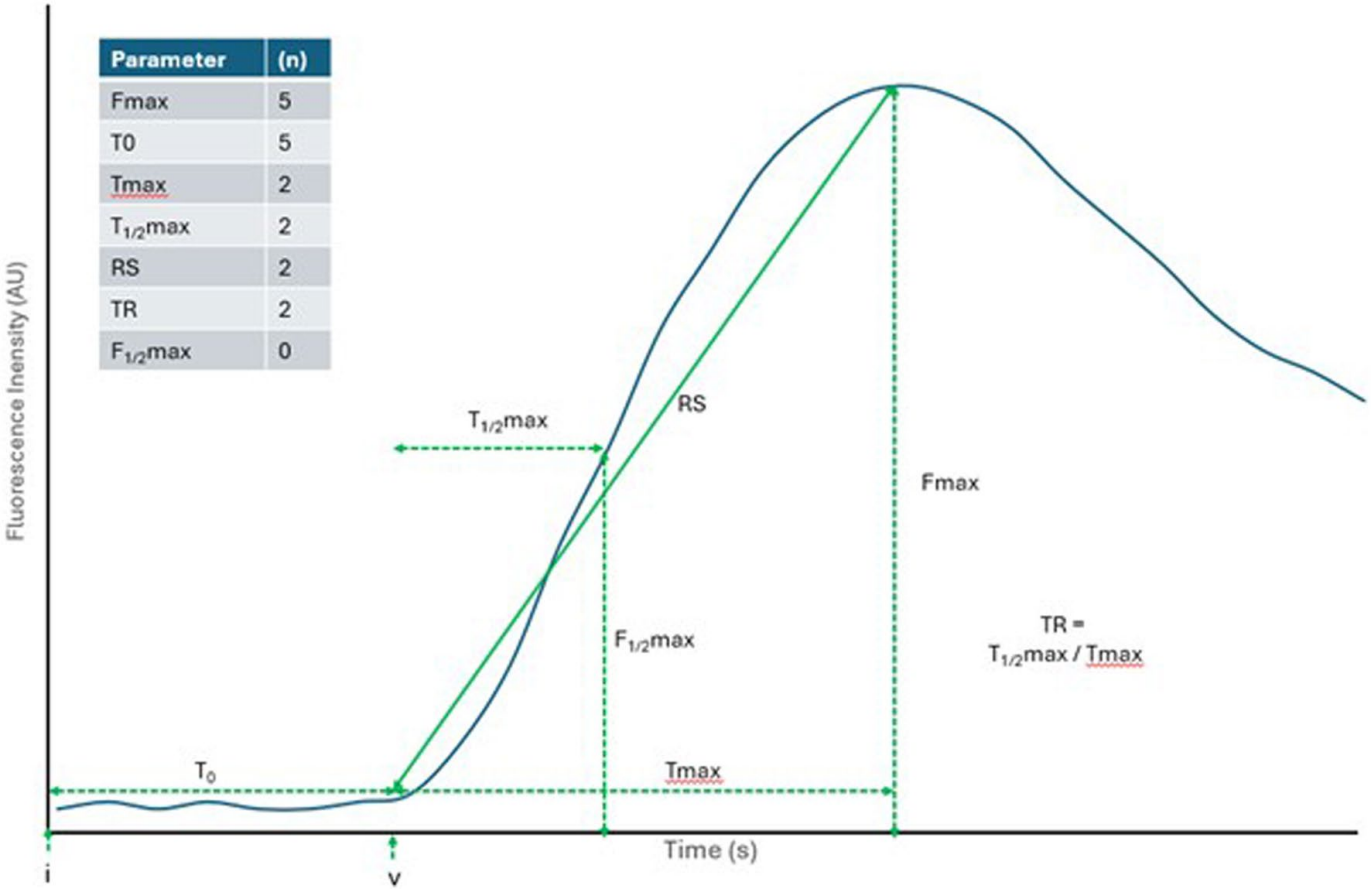
Schematic representation of the most commonly utilised perfusion parameters. AU arbitrary units, s seconds, i time of ICG injection, v time of first visible fluorescence, T0 time from ICG injection to first visible fluorescence, Fmax maximum intensity, Tmax time to reach maximum intensity from beginning of visible fluorescence, F1/2max half maximum intensity, T1/2max time to reach half maximum intensity from beginning of visible fluorescence, TR time ratio, (*n*) number of studies where parameter was statistically significantly correlated to endpoint

In total, in included studies, 26 different perfusion parameters were defined and used being broadly categorisable into intensity, inflow and outflow milestones (see Table 2 and Fig. 3 for details).

#### Intensity

Maximum fluorescence intensity (F_max_) was the second most frequently assessed parameter (*n* = 13). Other intensity parameters included half of the maximum intensity and the fluorescence intensity drop at 50 and 100 s after maximum intensity (*n* = 1 each).

#### Inflow

Time to reach maximum intensity from the beginning of visible fluorescence (T_max_) was the most frequently assessed parameter (*n* = 15). Time to reach half of maximum intensity from the beginning of visible fluorescence (T_1/2max_) and time from injection to visible fluorescence (T_0_) were also included in many studies (*n* = 8 and 7, respectively). Some of the calculated inflow parameters included time ratio (T_1/2max_/T_max_), relative slope (F_max_/T_max_), time from injection to reach maximum intensity (T_0_ + T_max_), maximum inflow slope (ingress slope), maximum inflow slope in percentage per second (normalised slope), rate of change and duration (timing) of the overall intensity curve, slope at ten seconds after visible fluorescence (RS_10s_).

#### Outflow

Only three of the included studies assessed parameters occurring after maximum fluorescence intensity. These were the maximum outflow slope (egress slope), area under the curve (AUC) in percentage after 30/60/120/180 s from T_max_ (AUC_30/60/120/180_), overall downslope, downslope at 50/100 s, centre of mass, the fluorescence difference between maximum intensity and intensity at a sharp drop then gentle decrease (F_plateau_), the time after maximum intensity F_plateau_ occurs (T_plateau_) and the decreased fluorescence slope (F_plateau_ divided by T_plateau_).

### Artificial intelligence (AI) models

Four studies used AI. Park et al. [32] developed an unsupervised machine learning model using a self-organizing map (SOM) network to predict the risk of anastomotic complications focussing only on rectal resections. This model was trained on 50 patient videos and tested on 15. The training dataset included 10,000 ICG curves extracted from 200 different locations within the fluorescence videos. The average intensity value was calculated from each region of interest for 40 s (at 30 frames/second). SOM clustering of the training data resulted in 25 ICG curve patterns and each pattern was risk-labelled (safe/intermediate/dangerous).

Dalli et al. [42] developed a patient calibrated quantitative ICGFA bowel transection recommender. Initially, a reference ICGFA curve was obtained from a 4-min ICGFA video recorded from the colon before any tissue dissection occurred. Subsequently, a second ICG dose was administered to obtain a determinative ICGFA curve following dissection and vessel ligation. The ICGFA imagery was tracked and quantified, producing ICG curves which underwent smoothing and normalisation. The reference curve was then scaled and shifted to match the determinative curve and if narrower or wider than the determinative curve, brisker or slower perfusion was inferred, respectively. A determinative curve with ≥ 85% match to the reference curve indicated sufficient perfusion. Consequently, the most distal region of interest, characterised by sufficient perfusion in the determinative ICGFA imagery, was defined and recommended as a safe transection site. The model was trained on 14 patient videos and validated on 9 patient videos.

Arpaia et al. [44] developed a machine learning model using the Feed Forward Neural Network algorithm (*Adam* optimizer*, cross-entropy* loss function) to classify whether perfusion was adequate or inadequate. In this, a region of interest (rectangle) was selected and divided into 20 vertical slices, and a histogram of the green band was generated. A fast-tracking algorithm was used to track these selected regions. The feature vector (20 vertical elements) was classified as either adequate or inadequate perfusion, with the correctness confirmed by the operating surgeons. The model was trained on eleven patient videos.

Singaravelu et al. [52] developed a deep learning bidirectional long short-term memory model to predict where an experienced ICGFA user would place the surgical stapler based on their interpretation of the imagery. ICGFA video frames underwent stabilisation and tracking. Training involved the selection of four lines of different lengths and positions across the fluorescent-nonfluorescent boundary of the region of interest, with the extraction of fluorescence parameters from multiple regions centred on each line. The site of stapling selected by the operating surgeon was then labelled “expert”, with the perfused side labelled “good” and the non-perfused side labelled “poor”. This model was trained on 25 patient videos and tested on 28.

## Clinical deployment and correlation

### Clinical deployment

In only one of the included studies were the perfusion parameters and quantification method actually used intra-operatively. Son et al. [34] (*n* = 68) developed a protocol based on tissue oxygen saturation (StO_2_) and T_1/2max_ with which they decided whether or not to change the intended transection line to a more proximal segment (this occurred in 6/68 cases). Three other studies performed real-time analysis of the ICGFA signal, but the operating surgeon was blinded to the interpretation of the quantification method in these cases. D’Urso et al. [40] (*n* = 22) used fluorescence-based enhanced reality (FLER) to superimpose the virtual perfusion cartography onto the bowel to display perfusion values with the aim of correlating this to intestinal lactates and mitochondrial respiration. Arpaia et al. [44] carried out real-time testing of their AI model in an unspecified number of cases with the aim of correlating to surgical classification of the region’s perfusion as adequate or inadequate. Singaravelu et al. [52] carried out real-time testing of their AI model with the aim of correlating to surgeon selection of the transection point. In the remaining studies, analysis of the ICGFA signal was conducted after the operation and so all intra-operative decisions made were based on the independent ICGFA interpretation of surgeon.

### Anastomotic complications

The most common outcome of interest in the studies was anastomotic complication rate, with eighteen studies reporting this and eleven of these assessing for correlation between perfusion parameters and anastomotic complication. Of the 26 parameters assessed, six were found to correlate with anastomotic complication rate. T_0_ and F_max_ were the most common (*n* = 4), with slope (*n* = 2), TR (*n* = 2), T_1/2max_ (*n* = 1) and T_max_ (*n* = 1) also found to correlate. The AI model generated by Park et al. [32] was more predictive of anastomotic complication than the perfusion parameters assessed (AUC 0.842 AI vs 0.734 TR and 0.750 T_1/2max_). Adams et al. [43] reported no correlation between the studied parameters and anastomotic complication, whilst Protyniak et al. [35], Faber et al. [39] and Meijer et al. [41] reported their study numbers as being too small to assess for correlation. When comparing studies with intra-corporeal ICGFA assessment versus extra-corporeal assessment, the study findings were similar. Han et al. [48] (intra-corporeal) reported F_max_ was lower in the AL group, in keeping the findings of Wada et al. [36] and Kim et al. [46] (extra-corporeal). Iwamoto et al. [49] (intra-corporeal) assessed the rectal stump after transection and found T_0_ was higher in the AL group, in keeping with the findings of Aiba et al. [47] and Hayami et al. [37] (extra-corporeal).

Five of the six studies focussing on rectal resections assessed for parameter correlation with anastomotic complication. In these, T_0_, F_max_ and TR were most found to correlate (*n* = 2), with T_1/2max_ also correlating in one study [34].

### Tissue oxygen saturation

Three studies [34, 50, 51] assessed for correlation between perfusion parameters and tissue oxygen saturation and in these, T_1/2max_, T_max_, T_0_ and F_max_ (all *n* = 1) were found to correlate with the former two correlating in one study specifically including rectal resections. Two of the studies utilised a hyperspectral imaging camera to assess tissue oxygen saturation [34, 51] and the third study utilised near-infrared spectroscopy [50].

### Metabolic markers

D’Urso et al. [40] reported that T_max_ correlated with both capillary lactates and mitochondrial respiration. Dalli et al. [42] reported correlation of lactate ratio with both downslope and F_max_, however the generated model did not correlate.

### Surgeon interpretation

Whilst thirteen studies reported the rate with which the surgeon changed the surgical plan with ICGFA, only one correlated this with measured parameters. Adams et al. [43] reported that in cases where the surgeon changed the surgical plan, the F_max_ was significantly lower and T_max_ significantly higher than in cases with no change. The AI model generated by Arpaia et al. [44] reported a prediction accuracy of 99.9% when comparing the perfusion of the selected region of interest versus surgeon classification as adequate or inadequate. Three studies assessed whether the quantification method matched the transection point selected by the operating surgeon. Bornstein et al. [38] reported disparity between their system and the selected transection point in 11 of 72 cases, whilst Dalli et al. [42] found their system matched the selected transection point in 10 of 11 bowel segments (9 patients). Singaravelu et al. [52] reported 85% accuracy at pixel level versus expert surgeon interpretation in the overall set of 28 unseen cases and 89% accurate for the 15 real-time testing cases. Kudszus et al. [12] did not report any correlation of perfusion parameters and outcomes (Table 3).

**Table 3:** Clinical endpoints and results

Paper	Change of mind	Anastomotic complication (AC)	Other	Results
Park et al. (2020)	3/50 training, 3/15 testing based on surgeon	3/50 leak, 1/50 stricture in training, 0 complications in testing		ROC curves of T_1/2max_, TR and AI model predictive of anastomotic complication risk by hypoperfusion (p 0.034, 0.006 and < 0.001, respectively)
Son et al. (2019)	7/86 based on surgeon	6/86 anastomotic complications		TR > 0.6 associated with anastomotic complication (*p* = 0.002)
Son et al. (2023)	6/68 based on readings	5/68 anastomotic complication 1/6 in change group	Hyperspectral Imaging using TIVITA Tissue System to measure StO_2_	T_1/2max_ ≤ 10 s associated with StO_2_ ≥ 60% (p < 0.01). T_max_ ≤ 30 s associated with StO_2_ ≥ 60% (p0.01)
Protyniak et al. (2015)	4/77 based on surgeon	2/77 leaks		Too small to assess for correlation
Wada et al. (2017)	18/112 based on surgeon	5/112 leaks		F_max_ < 52AU 100% sensitive, 92.5% specific, 38.5% PPV, 100% NPV for prediction of AL. Slope < 2.1AU/sec 100% sensitive, 75.7% specific, 16.1% PPV, 100% NPV for prediction of AL
Hayami et al. (2019)	1/22 based on surgeon	3/22 leaks		T_0_ significantly higher in AL group (*P* < 0.001)
Kudszus et al. (2010)	28/201 based on surgeon	7/201 leaks		NR
Bornstein et al. (2018)	NR	NR	Disparity between system and surgeon selection of transection point	11/72 disparity. 6/11 disparity of both perfusion and timing, 5/11 had single disparity
Faber et al. (2023)	4/20 based on surgeon	4/20 leaks	Perfusion patterns 1, 2 and 3	Too small to assess for correlation
D'Urso et al. (2021)	3/22 based on surgeon	5/22 leaks	Capillary lactates and mitochondrial respiration	T_max_ significantly higher at proximal resection site in AL group (*p* = 0.01)
Meijer et al. (2021)	1/4 based on surgeon	1/4 leaks		Too small to assess for correlation
Dalli et al. (2024)	NR	1/23 leaks	Surgeon selection of transection point and mean lactate concentrations	19 segments for development, 11 segments for validation. Region of interest-based recommendation matched surgeon selection in 10 of 11 cases in validation series. Moderate negative correlation with downslope and lactate ratio (*p* < 0.001). Moderate negative correlation with F_max_ and lactate ratio (*p* < 0.001)
Adams et al. (2024)	7/89 based on surgeon	9/85 leaks	Surgeon preserves bowel (accepted perfusion, AP) or clinical decision to resect back to healthy margin (rejected perfusion, RP)	No correlation with parameters and anastomotic leak. F_max_ lower in RP group (*p* = 0.03). T_max_ higher in RP group (*p* < 0.01)
Arpaia et al. (2022)	NR	NR		Prediction accuracy of 99.9% to assess if the quality of perfusion is adequate or inadequate
Gomez-Rosado et al. (2022)	16/69 based on surgeon	9/69 leaks		F_max_ significantly lower in AL group (*p* = 0.03). Slope significantly lower in AL group (*p* = 0.03)
Kim et al. (2017)	NR	2/310 leaks, 11/310 strictures		T_0_ significantly higher in stricture group (*p* = 0.01). F_max_ significantly lower in stricture group (p0.002)
Aiba et al. (2021)	2/110 based on surgeon	6/110 leaks	66 in marginal flow group, 44 in direct flow group	T_0_ significantly higher in AL in marginal flow group (*p* = 0.046)
Han et al. (2022)	NR	2/22 leaks	High and low ligation of the inferior mesenteric artery, 11 in each group	F_max_ significantly lower in overall AL group (*p* = 0.012)
Iwamoto et al. ((2020)	NR	6/25 leaks		T_0_ significantly higher in AL group (*p* = 0.03)
Egi et al. ((2022)	NR	0/12	INVOS, Medtronic to measure StO_2_	Strong correlation with T_0_ and StO_2_ (p = 0.017)
Soares et al. ((2022)	NR	NR	Multispectral imaging using SpectroCam, Ocean Insight to measure StO_2_	Strong correlation between F_max_ and StO2 (*p* < 0.001)
Singaravelu et al. ((2024)	3/53	NR	Surgeon selection of transection point	Deep learning model 85% accurate at pixel level versus surgeon selection

*NR* not reported, *ROC* receiver operating characteristic, *AI* artificial intelligence, *TR* time ratio, *StO*_*2*_ tissue oxygen saturation, *AU* arbitrary units, *PPV* positive prediction value, *NPV* negative prediction value

## Discussion

RCTs and meta-analyses report benefit of ICGFA in colorectal surgery amongst regular users, without the need for quantification methods, in relation to post-operative AL rates [3, 19–23]. With widespread availability of NIR-capable platforms, the use of ICGFA for perfusion assessment during colorectal surgery may so become standard of care as professional society guidelines assimilate such level 1 evidence irrespective of individual unit perception [53]. Potential barriers to broad ICGFA implementation include the associated learning curve [24–26], along with a lack of standardised training pathways and any associated cognitive load with adding a step into already complex procedures [54] as well as need to document correct interpretation. Real-time quantification of the ICGFA signal has been proposed as a method to offset these issues, especially for new adopters [55].

This review clarifies the current state of the published art regarding quantification with a focus on useful clinical deployment. Overall, experience remains at developmental stage with significant heterogeneity of study quality, methodology and results. Whilst all the studies use a commercially available NIR-capable platform, the outputs from these platforms are variable (and all perform variable signal processing in doing so) and so each quantification method has been tailored to individual imagers. Even in studies utilising the same imaging platform, different computational approaches mean discrepancies in ICGFA quantification that limit generalisability [56]. Furthermore, whilst fluorescence signal intensity varies significantly with factors such as camera lens configuration (0 degrees versus 30 degrees) and distance from as well as position of target (i.e. central or peripheral placement in the field of view [57]), only some studies standardise set-up protocols (although some work suggests standardisation may worsen signal computational inference [58]). Reflective perhaps of variance in clinical practice [59], significant heterogeneity is also observed regarding ICG dosing.

Whilst experiences to date demonstrate that quantification of the ICGFA signal is feasible, there is still uncertainty as to its value and purpose, and there remains no fully validated system. Only a minority of studies focus on rectal resections which is where the main (and perhaps only) benefit of ICGFA is supported by clinical trialling [21]. Anastomotic complication was the endpoint most commonly assessed with half of studies attempting to correlate the quantified parameters with this, with others targeting alternative surrogates such as tissue oxygen saturation (despite this itself being in need of clinical validation^1^ [34], and measurement standardisation), metabolic markers or novel imaging methods such as hyperspectral imaging [60]. Perhaps more meaningful and more achievable for clinical validation (given that ICGFA is now known to associate with reduced AL rates when used by ICGFA-experienced surgeons) are the quantitative methods grounded in representing expert interpretation.

Another issue with ICGFA quantification may be the focus on seemingly separate fluorescence parameters although these are naturally interrelated as a time series. Whilst appearing distinct metrics for characterising fluorescence behaviour, they reflect connected dynamic, physical processes and their measurement may change with altering measurement conditions. AI methods [34, 42, 44, 52] may provide better analytics for this and its use seems likely to increase in this field and indeed others [61, 62]. Although uncommon as an endpoint as yet, ICGFA interpretation seems apposite for AI systems as the foundational phenomena are understandable even if the specifics of deep learning methods that exploit them may not be.

Aside from uncertainty of quantification method of best fit, there are other challenges to developing clinical commercial systems. Whilst it does appear that small datasets are sufficient to create and train quantification models, for validation and testing larger volumes of patient data from diverse populations is necessary, and there is no existing assessable warehouse of annotated imagery. Whilst individual units may hold some libraries, current data protection regulations make sharing this data with third parties challenging, meaning development studies need to perform their own prospective validations, including in different centres. Furthermore, there is concern at regulatory level about the level of decision support provided as this has liability impact. This concern could possibly be minimised utilising the quantitative method grounded in expert surgeon interpretation as it could, if proven to be validated in clinical studies, provide the user with the information of how an experienced ICGFA user would interpret the imagery [63]. Lastly, the optimum method for initial deployment is uncertain as introduction of algorithms into imager camera heads or towers may impact existing regulatory approvals, and so any new potential systems need deployment in a separable device as seen currently often in endoscopic polyp identification methods [64, 65]. These factors may account for why ICGFA quantification is still predominantly in the development phase (only four studies in this review have managed to perform real-time quantification of the ICGFA signal with only one of these using the quantification to inform intra-operative surgical decision making) in contrast to the use of ICGFA which has progressed to the point of seven published RCTs comparing the use of ICGFA to non-use [1–4, 18, 23, 66], with others now completing enrolment/analysis [67–69].

In terms of study limitations, as discussed above, the biggest issue is the variability in study type which limited formal aggregation and meta-analysis. It is possible of course too that commercial entities developing systems are progressing without academic disclosure of their method and efforts.

## Conclusion

This review demonstrates that quantification of the ICGFA signal during colorectal surgery appears confined to academic explorations and the absence of deployed methods that would enable methodologically appropriate clinical trialling highlights a critical gap in the current clinical literature. ICGFA can be used reliably with experience by observation alone, computational methods may usefully aim to replicate this evaluation by representing experience user interpretation rather than aiming to simply provide further ungrounded data for additional interpretation.
